# Bilateral Accessory Plantaris Muscles With Variant Origins and Insertions: A Case Report

**DOI:** 10.7759/cureus.103914

**Published:** 2026-02-19

**Authors:** Christian M Furno, Jack W Kauffman, Vhuthuhawe Madzinge

**Affiliations:** 1 Department of Biology and Chemistry, Liberty University, Lynchburg, USA

**Keywords:** accessory plantaris muscle, achilles tendinopathy, bilateral variation, fool's nerve, nerve block, plantaris muscle, plantaris tendon, variant origin and insertion

## Abstract

The plantaris muscle typically originates at the lower portion of the lateral supracondylar line of the femur and oblique popliteal ligament of the knee joint and inserts medial to the Achilles tendon at the calcaneal tuberosity. Functionally, this muscle is unclear, as it could be for proprioception of the leg or flexion of the knee and ankle. Additionally, it is absent in some individuals. If present, the tendon is often mistaken for the tibial nerve, which is why it is known as the “fool’s nerve.” This case study involves the discovery of a bilateral plantaris muscle duplication in an 88-year-old Japanese woman during a routine cadaveric dissection. This deviates from the more common unilateral presence of an accessory plantaris muscle that is predominantly reported in previous studies. Additionally, both the accessory origin and insertion differ between the two. The right accessory muscle belly originated lateral to the primary, and the tendon terminated in the tendon of the lateral head of the gastrocnemius. Conversely, the left accessory muscle belly originated superomedial to the primary muscle, where the corresponding tendon terminated in the tendon of the medial head of the gastrocnemius. Anatomical variations, including duplication, of the plantaris muscle remain an area of ongoing research due to its clinical relevance with Achilles tendinopathy, tendon grafts, and surgeries. This case reports bilateral asymmetrical accessory plantaris muscles and tendons, which can alert physicians to avoid anesthetic complications when performing popliteal or tibial nerve blocks.

## Introduction

The plantaris muscle is a small, thin muscle that joins the gastrocnemius and soleus muscles to form the triceps surae muscle group [1]. All three muscles are innervated by the tibial nerve and constitute the bulk of the calf. Physiologically, this muscle is considered vestigial as a flexor [2,3]. However, due to the high density of muscle spindles observed in the muscle belly, its functionality is thought to be proprioceptive instead of mechanical. It acts as a monitor for muscle stretch, tension, and position of the larger calf muscles. Additionally, it may help indicate position within the Achilles tendon complex during the eccentric loading involved in running or descending a flight of stairs [4]. Furthermore, this muscle shows stable polymorphism, meaning that it does not regress due to disuse in adulthood, therefore affirming the idea of proprioception rather than flexion. Anatomically, the plantaris originates at the lower portion of the lateral supracondylar line of the femur and oblique popliteal ligament of the knee joint, then joins the posterosuperficial compartment of the calf [5]. In most cases, its long tendon courses alongside the calcaneal tendon in an inferomedial direction toward the medial crural region between the medial gastrocnemius and soleus muscles, inserting just medial to the Achilles tendon at the calcaneal tuberosity [4]. Classically, its long tendon, slender appearance, and proximity to the Achilles tendon cause many first-year medical students to confuse the plantaris tendon for the tibial nerve, thus giving it the name “freshman nerve,” while it is also commonly known in medical literature as the “fool’s nerve” [5,6]. Its nerve-like appearance is noted by clinicians when performing nerve blocks and diagnostic imaging. Previous literature has noted that anomalous muscles such as these may be mistaken for a neoplasm, which leads to unnecessary imaging, biopsies, and even surgery [6]. This anatomical variance is particularly relevant not only for diagnostic purposes but also for its potential involvement in hindfoot disturbances in clinical presentations of Achilles tendinopathy and tennis leg in athletes and middle-aged individuals [6-10]. According to Herzog, the unilateral presence of accessory plantaris muscles had a 6.3% incidence in a study consisting of 1000 patients [8]. Conversely, we describe a case report in which we observed a bilateral duplication of the plantaris muscle that was asymmetrical in its duplicate origin and insertion in a routine cadaveric dissection.

## Case presentation

**Figure 1:**
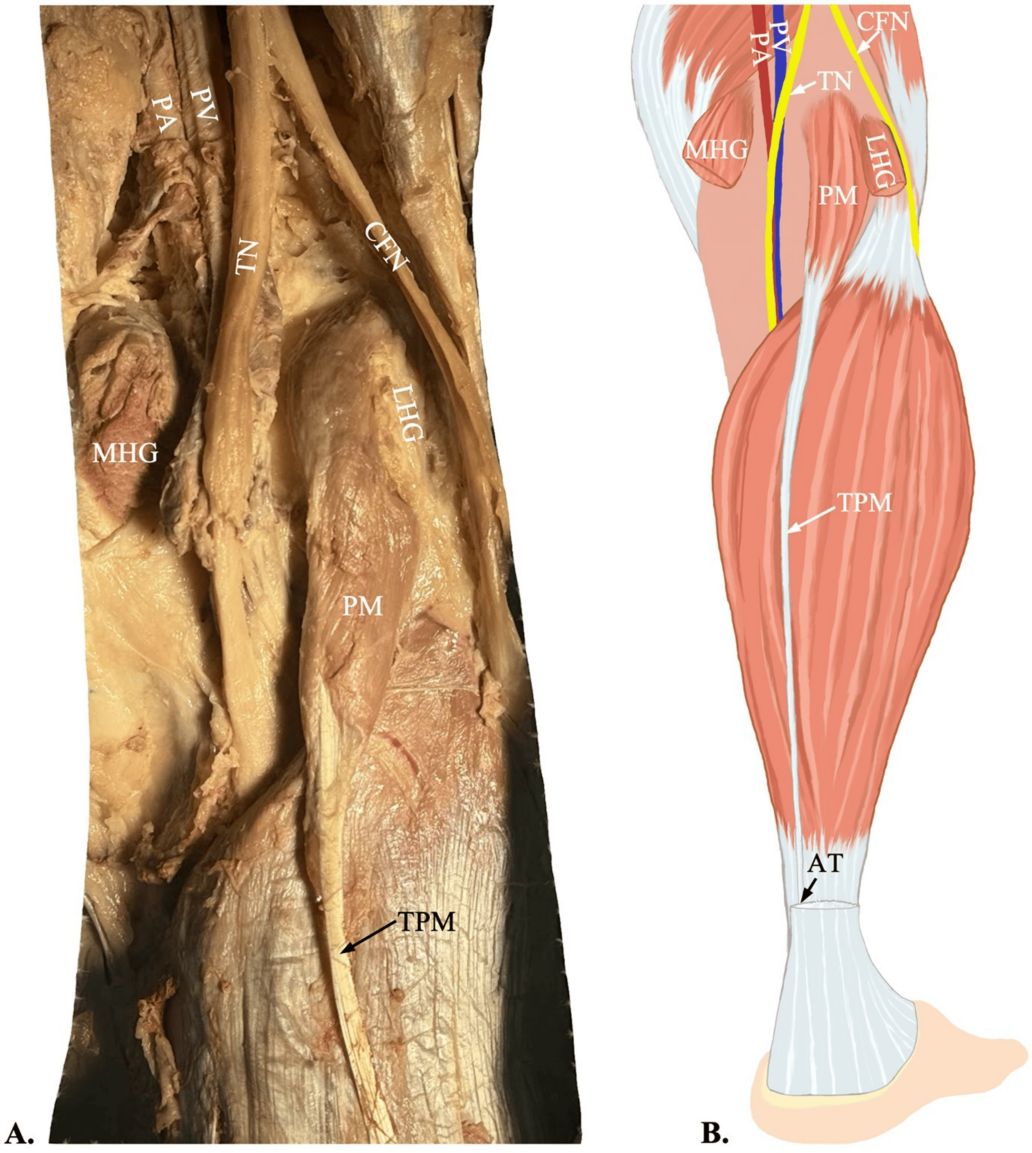
Origin and insertion of the plantaris muscle A: Dissection of the right popliteal fossa shows the plantaris muscle (PM) originating on the lateral supracondylar line of the femur and forming the tendon of the plantaris muscle (TPM). The medial head of the gastrocnemius muscle (MHG) and lateral head of the gastrocnemius muscle (LHG) have been cut to display the plantaris muscle origin. B: An illustration of the right leg showing the course of the tendon of the plantaris muscle merging into the Achilles tendon (AT). Figure B was created by author Jack Kauffman using Sketchbook, Inc., San Francisco, California. PA: popliteal artery; PV: popliteal vein; TN: tibial nerve; CFN: common fibular nerve

**Figure 2:**
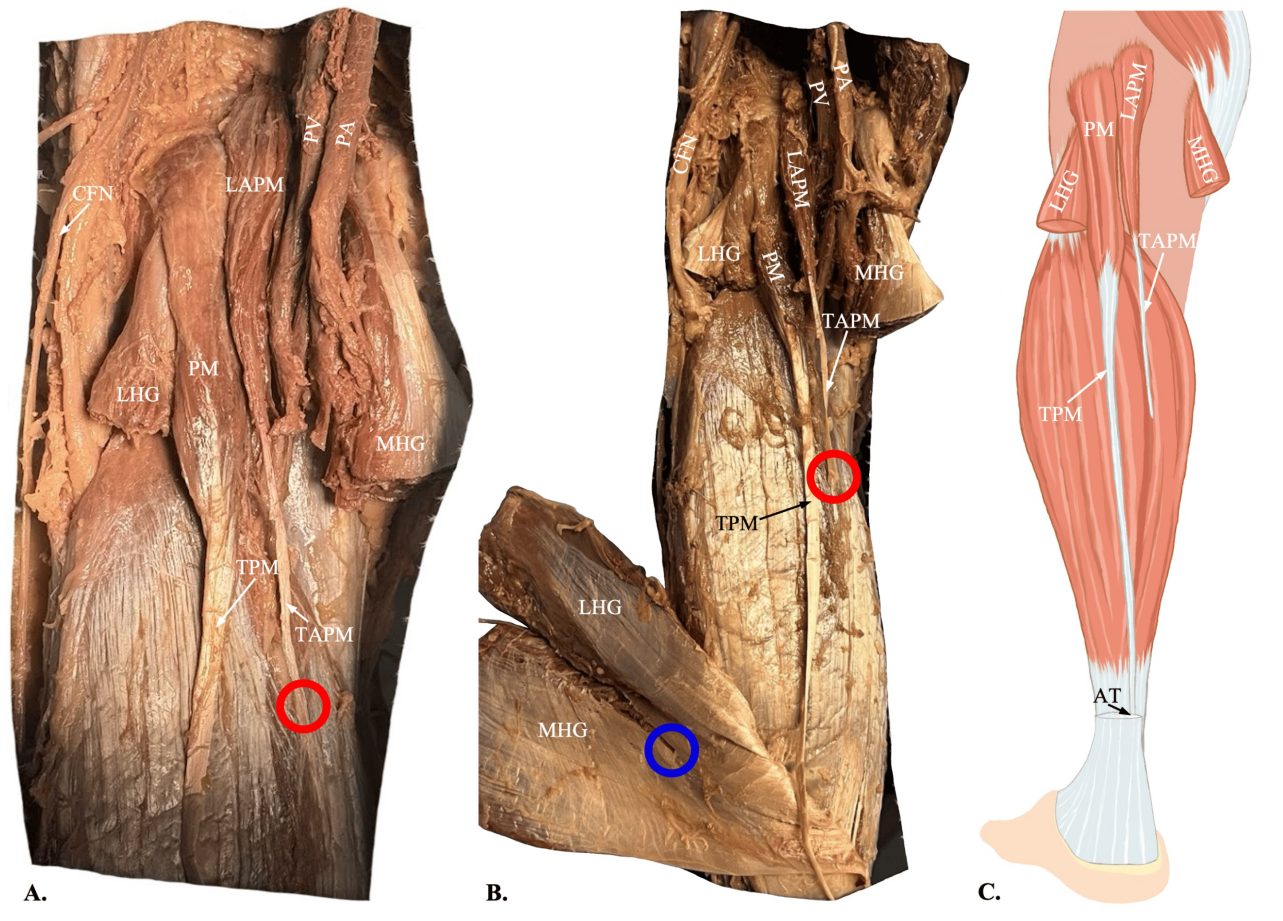
Origin and insertion of the left accessory plantaris muscle A: Dissection of the left popliteal fossa shows an accessory plantaris muscle (LAPM) originating superomedially to the origin of the primary plantaris muscle (PM). The origins of the two muscle bellies are found at the superior aspect of the intercondylar fossa of the femur and the lateral supracondylar line of the femur, respectively. The LAPM forms the tendon of the accessory plantaris muscle (TAPM), which has been cut (red circles) to reflect the gastrocnemius muscle. The medial head of the gastrocnemius muscle (MHG) and lateral head of the gastrocnemius muscle (LHG) have been cut to display the plantaris muscle’s origins. B: A more distal view of the dissection shows the gastrocnemius reflected laterally to reveal the insertion of the TAPM into the MHG (blue circle). C: An illustration of the left leg shows the course of the tendon of the plantaris muscle (TPM) merging into the Achilles tendon (AT) and the TAPM terminating at the level of its insertion into the MHG. Figure C was created by author Jack Kauffman using Sketchbook, Inc., San Francisco, California. PA: popliteal artery; PV: popliteal vein; CFN: common fibular nerve

**Figure 3:**
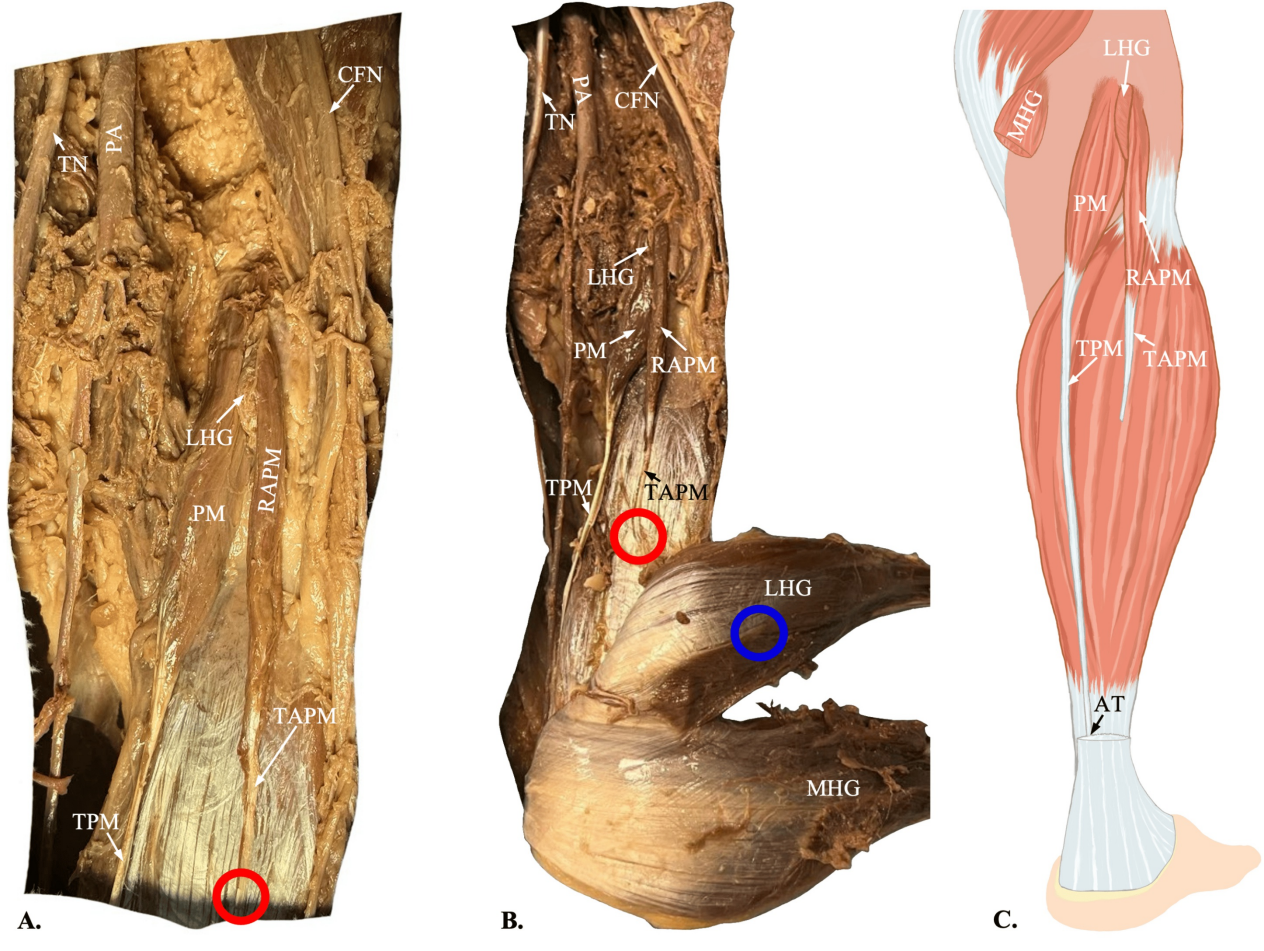
Origin and insertion of the right accessory plantaris muscle A: Dissection of the right popliteal fossa shows an accessory plantaris muscle (RAPM) originating just medial to the origin of the primary plantaris muscle (PM), with the lateral head of the gastrocnemius (LHG) interposing between the two. The origins of the two muscle bellies are found at the medial and lateral aspects of the supracondylar line of the femur, respectively. The RAPM forms the tendon of the accessory plantaris muscle (TAPM), which has been cut (red circles) to reflect the gastrocnemius muscle. The medial head of the gastrocnemius muscle (MHG) and the LHG have been cut to display the plantaris muscles’ origins. B: A wider view of the dissection shows the gastrocnemius reflected laterally to reveal the insertion of the TAPM into the LHG (blue circle). C: An illustration of the right leg shows the course of the tendon of the plantaris muscle (TPM) merging into the Achilles tendon (AT) and the TAPM terminating at the level of its insertion into the LHG. Figure C was created by author Jack Kauffman using Sketchbook, Inc., San Francisco, California. PA: popliteal artery; PV: popliteal vein; TN: tibial nerve; CFN: common fibular nerve

During a routine cadaveric dissection of the posterior legs of an 88-year-old Japanese female, undergraduate anatomy students found anomalous accessory plantaris muscles bilaterally. However, as photographed and documented, the anomalies were asymmetrical in their origin and insertion. The primary plantaris muscle bellies in both legs originated at the lateral supracondylar line of the femur, and the tendons inserted just medially into the Achilles tendon (Figure 1). The origin of the left accessory plantaris muscle belly was found superior and deep to the gastrocnemius heads in the popliteal fossa, relative to the normal muscle. The accessory tendon terminated in the tendinous portion of the medial head of the gastrocnemius muscle. The right accessory plantaris muscle belly appeared to be a duplicate head of the primary, originating just lateral to the lateral supracondylar line of the femur. The corresponding accessory tendon is inserted into the tendinous portion of the lateral head of the gastrocnemius muscle. Both accessory muscles had noticeably thinner muscle bellies and tendons than the primaries, while they still presented a fleshy appearance composed of several muscle spindles. Additionally, both the primary and accessory appeared to be innervated by the tibial nerve and supplied with blood by the popliteal artery (Figures 2, 3).

## Discussion

**Figure 4:**
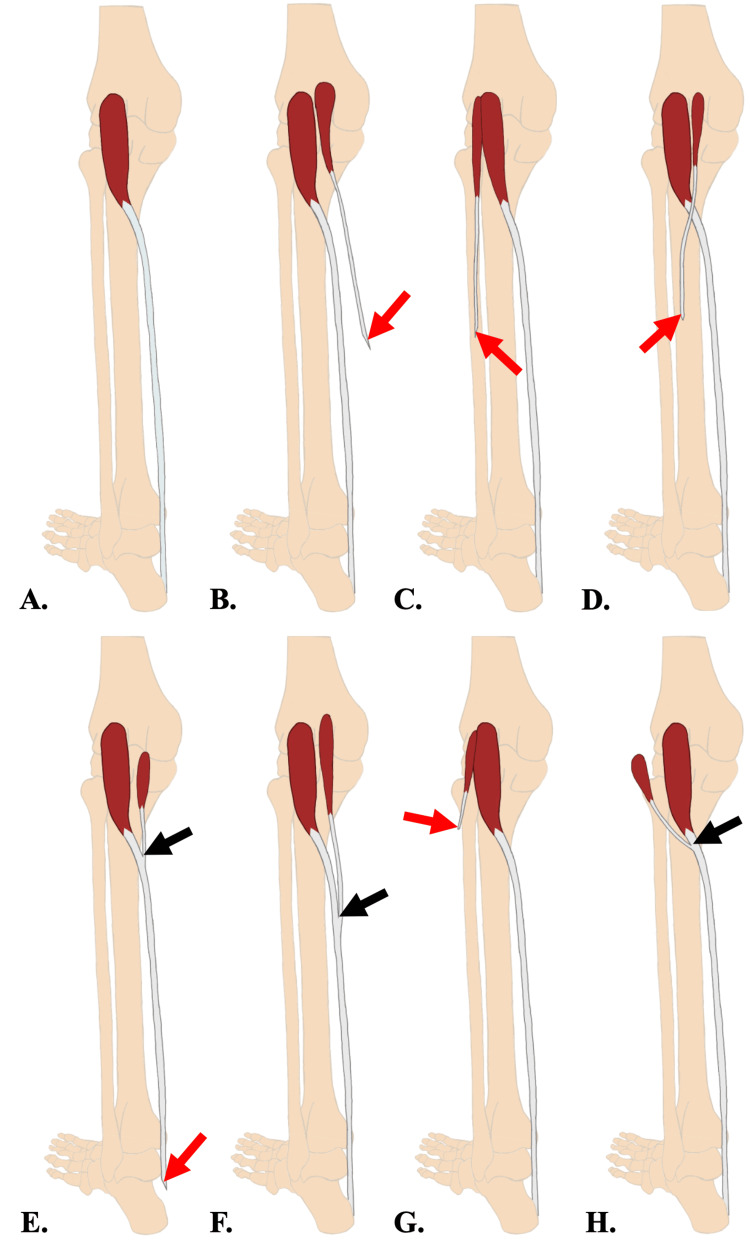
Comparative illustrations of accessory plantaris muscle variations from the current study and previous studies Previous studies showed variations of the accessory plantaris muscle’s origin and insertion [6,11-16]. Refer to Table 1 for more information about their origins and insertions. The figure was created by author Jack Kauffman using Sketchbook, Inc., San Francisco, California. A: none - no accessory plantaris muscle; B: medial head of the gastrocnemius muscle; C: lateral head of the gastrocnemius muscle; D: aponeurosis of the gastrocnemius muscle; E: flexor retinaculum of the ankle via the common plantaris tendon; F: calcaneus bone via the common plantaris tendon; G: iliotibial band; and H: calcaneus bone via the common plantaris tendon. Red arrows: tendons of the accessory plantaris muscle or common plantaris tendons terminate at the level of their insertion; Black arrows: tendons of the primary and accessory plantaris muscles join to form the common plantaris tendon *For visual consistency and ease of comparison, all variations were represented on the left leg regardless of the actual leg on which the study found them.

Accessory muscles are known sources of misidentification in clinical practice. In addition to its absence in 7-20% of the population, there is frequent variation in muscle origin, tendon insertion, and duplication [4]. The tendon of the plantaris muscle, although it is small and deep in the lower leg, warrants attention by physicians, especially in cases of diagnostic imaging and pre-surgical nerve blocking. The previously reported cases of unilateral presence of accessory plantaris muscles will present challenges even to physicians who already acknowledge and avoid the plantaris tendon during anesthesia administration [11]. In such a scenario, an anesthesiologist, after identifying and mentally ‘eliminating’ the primary plantaris tendon, could incorrectly conclude that the accessory tendon is the tibial nerve rather than an anomalous tendon. Additionally, although the origin of the accessory plantaris muscle is relatively consistent at the lateral supracondylar line, the variation in accessory tendon insertion is vast (Figure 4, Table 1). This case adds another layer of complexity as there is a bilateral presence of accessory plantaris muscles, which also differ from one another in origin and insertion. Therefore, variations such as what is reported in this case will provide greater awareness of a challenge that is already convoluted. While it is difficult to differentiate between the tendon and tibial nerve, a cross-sectional MRI can prove useful for radiologists seeking to eliminate other possibilities [8]. Through this tool, the accessory muscle would show the well-defined architecture of a muscle and tendon rather than the rounded or irregular appearance of a soft tissue mass. On a different yet clinically relevant note, if the accessory tendon of the plantaris muscle is properly identified, it could prove useful in orthopedic operations requiring tendon grafts, such as hand flexor tendon reconstruction, atrioventricular valve repair, etc. [11-13]. 

**Table 1:** Origins and insertions of primary and accessory plantaris muscles The letters A-H correspond with Figure 4.

	Plantaris Muscle Head	Origin	Insertion
Normal Anatomy (A)	–	Lateral supracondylar line of the femur	Calcaneus bone
	–	–	–
Left Leg (B) (Current Study)	Primary	Lateral supracondylar line of the femur	Calcaneus bone
	Accessory	Superior aspect of the intercondylar fossa of the femur	Medial head of the gastrocnemius muscle
Right Leg (C) (Current Study)	Primary	Lateral supracondylar line of the femur	Calcaneus bone
	Accessory	Lateral supracondylar line of the femur	Lateral head of the gastrocnemius muscle
Maggard et al. (D) [11]	Primary	Lateral supracondylar line of the femur	Calcaneus bone
	Accessory	Lateral aspect of the intercondylar fossa of the femur	Aponeurosis of the gastrocnemius muscle
Meyur et al. (E) [14]	Primary	Lateral supracondylar line of the femur	Flexor retinaculum of the ankle via the common plantaris tendon
	Accessory	Medial aspect of the oblique popliteal ligament	Flexor retinaculum of the ankle via the common plantaris tendon
Kwinter et al. (F) [15]	Primary	Lateral supracondylar line of the femur	Calcaneus bone via the common plantaris tendon
	Accessory	Superior aspect of the intercondylar fossa of the femur	Calcaneus bone via the common plantaris tendon
Herzog et al. (G) [6]	Primary	Lateral supracondylar line of the femur	Calcaneus bone
	Accessory	Lateral supracondylar line of the femur	Iliotibial band
Futa et al. (H) [16]	Primary	Lateral supracondylar line of the femur	Calcaneus bone via the common plantaris tendon
	Accessory	Iliotibial band	Calcaneus bone via the common plantaris tendon

## Conclusions

This discovery of a bilateral accessory plantaris muscle with varying origins and insertions, to our knowledge, is one of the first of its kind. Therefore, it prompts a meta-analysis of its prevalence in the population group from which we obtained the cadaveric donors in conjunction with previously reported cases. In the clinic, there continues to be ongoing discussion of the role this plays in Achilles tendinopathy, tennis leg, and so forth. Ultimately, awareness of accessory plantaris muscles will potentially prevent mistakes during anesthesia administration to the leg. Given that the incidence rate of the accessory plantaris muscle is increasing, it is imperative that clinicians familiarize themselves with the possibility of coming across an accessory plantaris muscle as well as its variants.
